# Supplementary material for: Computational Design of a Chimeric Vaccine against Plesiomonas shigelloides Using Pan-Genome and Reverse Vaccinology
Source: Vaccines (Basel). 2022 Nov 8;10(11):1886. doi: 10.3390/vaccines10111886 (PMC9697808; doi:10.3390/vaccines10111886)
Supplement: Supplementary file 1 [file vaccines-10-01886-s001.zip › vaccines-1948878-supplementary.pdf]

SUPPLEMENTARY FILES

Table S1. Physiochemical properties of proteins. Number of amino acid, molecular weight (M.W), Theoretical PI (T.PI), Instability index, aliphatic index, and Grand Average of Hydropathy (GRAVY).

Protein ID	Amino acid	M. Weight	T.PI	Instability Index	Aliphatic Index	GRAVY
>core/789/1/Org1_Gene3342	431	45.82076	4.97	19.14	72.88	-0.424
>core/1292/1/Org1_Gene1910	338	37.03311	4.9	27.92	77.9	-0.39
>core/2917/1/Org1_Gene957	133	13.95765	5.44	37.56	93.91	-0.169
>core/203/1/Org1_Gene311	688	75.19106	6.25	33.06	99.56	-0.197
>core/205/1/Org1_Gene3200	686	75.93728	5.76	33.94	73.8	-0.535
>core/378/1/Org1_Gene1902	560	60.80733	6.64	46.7	91.54	-0.189
>core/627/1/Org1_Gene1181	469	51.33913	5.65	44.78	99.55	-0.162
>core/1191/1/Org1_Gene230	356	38.72262	5.91	26.35	71.88	-0.355
>core/1645/1/Org1_Gene1459	290	33.38216	9.08	41.43	84.07	-0.451

>core/1892/1/Org1_Gene2068	251	28.18009	9.01	29.38	79.52	-0.41
>core/391/1/Org1_Gene2768	553	61.70417	5.35	28.72	76	-0.539
>core/558/1/Org1_Gene1999	488	54.88054	5.76	32.07	71.21	-0.517
>core/1117/1/Org1_Gene2629	368	40.63403	6.03	56.85	84.78	-0.22
>core/1152/1/Org1_Gene351	364	37.90081	9.51	30.4	105.27	0.18
>core/1281/1/Org1_Gene232	339	37.43379	6.05	29.99	92.39	-0.242
>core/1358/1/Org1_Gene2306	328	36.3557	7.89	60.96	70.34	-0.536
>core/1458/1/Org1_Gene1735	315	32.93791	9.16	37.31	103.9	-0.026
>core/1637/1/Org1_Gene1588	291	31.984	10.58	32.14	86.84	-0.3
>core/1838/1/Org1_Gene2194	258	27.11732	9.15	27.75	86.01	-0.117
>core/1915/1/Org1_Gene947	248	26.35666	5.25	34.27	77.98	-0.367
>core/1953/1/Org1_Gene1233	244	27.67492	8.94	46.57	88.73	-0.251
>core/2369/1/Org1_Gene482	193	21.35687	5.56	23.46	77.93	-0.21

Table S2. MHC alleles used in epitopes prediction.

MHC Molecules	Alleles
MHC-I	HLA-A*01:01, HLA-A*01:01, HLA-A*02:01, HLA-A*02:01, HLA-A*02:03, LA-A*02:03, HLA-A*02:06, HLA-A*02:06, HLA-A*03:01, HLA-A*03:01, HLA-A*11:01, HLA-A*11:01, HLA-A*23:01, HLA-A*23:01, HLA-A*24:02, HLA-A*24:02, HLA-A*26:01, HLA-A*26:01, HLA-A*30:01, HLA-A*30:01, HLA-A*30:02, HLA-A*30:02, HLA-A*31:01, HLA-A*31:01, HLA-A*32:01, HLA-A*32:01, HLA-A*33:01, HLA-A*33:01, HLA-A*68:01, HLA-A*68:01, HLA-A*68:02, HLA-A*68:02, HLA-B*07:02, HLA-B*07:02, HLA-B*08:01, HLA-B*08:01, HLA-B*15:01, HLA-B*15:01, HLA-B*35:01, HLA-B*35:01, HLA-B*40:01, HLA-B*40:01, HLA-B*44:02, HLA-B*44:02, HLA-B*44:03, HLA-B*44:03, HLA-B*51:01, HLA-B*51:01, HLA-B*53:01, HLA-B*53:01, HLA-B*57:01, HLA-B*57:01, HLA-B*58:01, HLA-B*58:01
MHC-II	HLA-DRB1*01:01, HLA-DRB1*03:*04:01,HLA-DRB101,HLA-DRB1*04:05,HLADRB1*07:01,HLADQA1*03:01/DQB1*03:02,HLADQA1*03:01/DQB1*03:02,HLADQA1*01:02/DQB1*06:02,HLADPA1*02:01/DPB1*01:01,HLADPA1*01:03/DPB1*04:01,HLADPA1*03:01/DPB1*04:02,HLADPA1*02:01/DPB1*05:01,HLADPA1*02:01/DPB1*14:01

Table S3 Predicted B- and T-cells (MHC-I and MHC-II) epitopes with their least percentile (P.rank) rank and Inhibitory concentration (IC_{50}) predicted score

B-Cell Epitope	MHC-I	P.rank	MHC-II	P.rank	MHcPred IC50 score
TVGFKESRAEFSDVYSSSIFSNPKTTVGG	GFKESRAEF	0.44	TVGFKESRAEFSDV	8	27.93

VQVPTEAGQPSASTKLDVGVNLPANQAAVTKTPFDFDDPDTYSKSTSTSIYDSLGNSYSMTT	VQVPTEAGQ	5.4	VQVPTEAGQPSAST	6.8	76.6
NFDKPTQYASDFNVKKINENGVVVGRL	KINENGVVV	0.29	NVKKINENGVVVG	1.4	71.12
ENKALSQETRSVA	ENKALSQETR	0.54	ENKALSQETRSVA	30	16.11
KQGYASANDETK	KQGYASANDE	19	KQGYASANDETK	11	4
TLDYRLNPTDSRW	RLNPTDSRW	0.04	TLDYRLNPTDSRW	3.5	5.28
	TLDYRLNPT	4.4			7.67
KTEFDEYRVTKKQSDKVD	RVTKKQSDK	0.29	DEYRVTKKQSDKV	6.4	22.59
EDKTQGEREGKNRPIPADGR	QGEREGKNRP	4.1	QGEREGKNRPIPAD	28	53.09
GRDKKTNQPLNN	RDKKTNQPL	0.99	GRDKKTNQPLNN	16	56.36

Table S4. Docking score of top 20 complexes of vaccine to MHC-I complexes generated by PatchDock server, energy is presented in kJ.mol-1.

Solution No	Score	Area	Atomic contact Energy
1	18650	2840.1	377.64
2	18118	3417.4	463.26
3	17826	2600.1	88.2
4	17808	2337.5	208.78
5	17454	2758.2	63.45
6	17114	2338.4	13.06
7	17012	2150.5	248.83
8	16946	2442	390.77
9	16820	2149.7	436
10	16786	2613.8	223.86
11	16770	2582.7	139.58
12	16756	2753	114.24
13	16626	2407.4	306.52
14	16568	1985	461.28
15	16564	2217.2	338.62
16	16556	2184.8	364.19
17	16540	2077.1	465.41
18	16532	2243.5	390.63
19	16454	2522.1	188.37
20	16408	2527.8	497.2

Table S5. Docking score of top 20 complexes of vaccine to MHC-II complexes generated by PatchDock server, energy is presented in kJ.mol-1

Solution No	Score	Area	Atomic contact Energy
1	20354	2881.8	-108.33
2	19610	3382.9	-63.11
3	19176	2978.7	-177.41
4	18106	2621.6	359.51
5	17934	3212	-116.73
6	17624	3191.7	128.84
7	17544	2806.8	74.39
8	17440	2912.1	497.06
9	16924	2357.9	281.2
10	16772	2449.5	84.71
11	16748	2416.1	294.47
12	16746	2218.7	411.07
13	16522	2184.5	-126.91
14	16180	2296.4	-97.58
15	16176	2454.5	36.94
16	16174	3291	-97.47
17	16102	2506.6	279.96
18	16082	2301.9	291.38
19	16064	2298.9	112.39
20	16030	2449.3	56.68

Table S6. Docking score of top 20 complexes of vaccine to TLR-4 complexes generated by PatchDock server, energy is presented in kJ.mol-1.

Solution No	Score	Area	Atomic contact Energy
1	20576	3063.7	298.48
2	20556	3417.9	376.86
3	18760	2784.6	215.91
4	18610	2539.2	380.55
5	17968	2960.7	98.43
6	17724	2666.5	397.88
7	17596	2531.5	224.04
8	17536	2955.9	469.72
9	17422	2625.5	280.88
10	17400	2291.7	181.33
11	17288	2421.5	349.11
12	17280	2382.2	254.79
13	16786	2874.8	455.55
14	16724	3323.4	371.08
15	16722	2268.4	-29.82
16	16584	2453.7	460.7
17	16576	2426.2	397.85
18	16564	2789.7	251.75
19	16530	2052	495.18
20	16472	2977.6	494.18

Table S7. Top 10 refined docked complexes of vaccine to MHC-I and model vaccine generated by FireDock server.

Rank	Solution Number	Global Energy	Attractive VdW	Repulsive VdW	ACE	HB
1	10	7.65	0	0	0	0
2	5	12.97	-7.05	2.25	4.33	-0.93
3	4	13.4	-39.01	39.96	9.4	-2.72
4	3	20.09	-3.89	0.29	4.63	0
5	8	23.53	-28.78	17.88	17.27	-2.32
6	6	39.18	-29.09	30.63	10.62	0
7	2	139.41	-16.54	164.74	9.07	0
8	7	157.51	-14.69	200.6	8.49	-2.16
9	9	288.52	-29.29	385.97	12.9	-4.99
10	1	346.28	-23.5	453.19	10.04	-2.5

Table S8. Top 10 refined docked complexes of vaccine to MHC-II and model vaccine generated by FireDock server.

Rank	Solution Number	Global Energy	Attractive VdW	Repulsive VdW	ACE	HB
1	8	3.11	-4.44	1.75	1.71	0
2	3	3.73	0	0	0.02	0
3	5	7.72	0	0	0	0
4	4	12.37	-6.09	9.67	4.28	-0.6
5	7	14.29	-6.85	3	2.38	0
6	9	1292.11	-35.93	1607.02	10.34	-4.42

7	10	2188.23	-63.68	2851.64	-1.73	-8.51
8	1	3656.2	-94.44	4778.14	-5.12	-9.92
9	6	4173.52	-98.16	5406.79	-0.29	-6.95
10	2	15953.26	-153.86	20260.04	0.08	-20.58

Table. S9. Top 10 refined docked complexes of vaccine to TLR-4 and vaccine generated by FireDock server.

Rank	Solution Number	Global Energy	Attractive VdW	Repulsive VdW	ACE	HB
1	4	-15.33	-22.6	7.55	2.29	-2.62
2	5	-10.4	-16.68	12	-1.46	-0.97
3	8	-2.46	-32.04	17.01	17.1	-3.91
4	7	-0.26	-9.48	3.69	3.74	0
5	9	2.21	-3.55	0	2.6	0
6	6	3.73	-4.83	0	3.25	-0.77
7	10	9.21	-7.31	3.8	3.22	-0.37
8	3	24.75	-23.79	9.73	14.64	-1.38
9	2	28.99	-10.56	1.38	6.8	-0.49
10	1	1424.61	-87.25	1939.19	16.72	-16.03

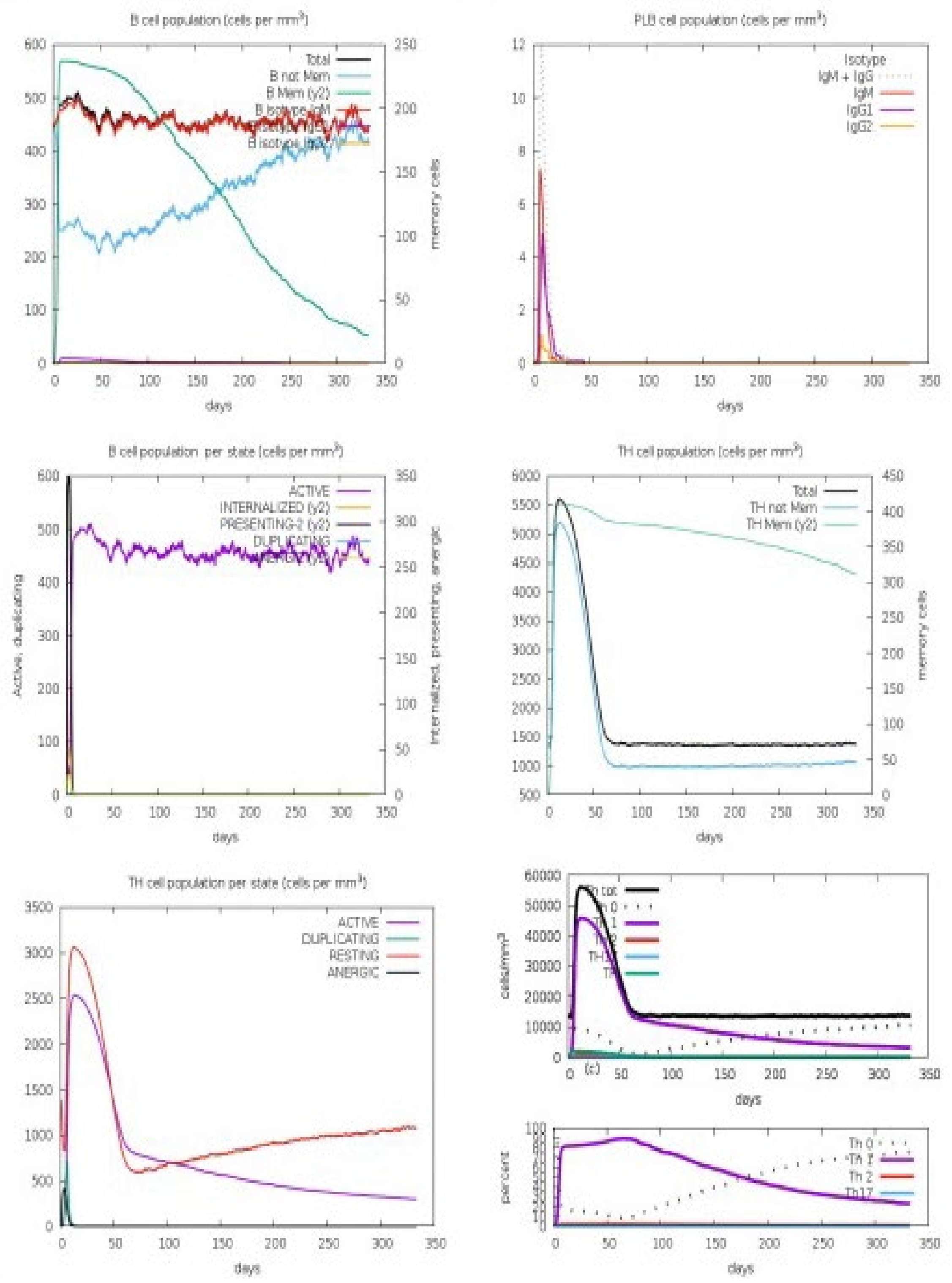

Figures S1. B-cell responses to the designed vaccine construct.

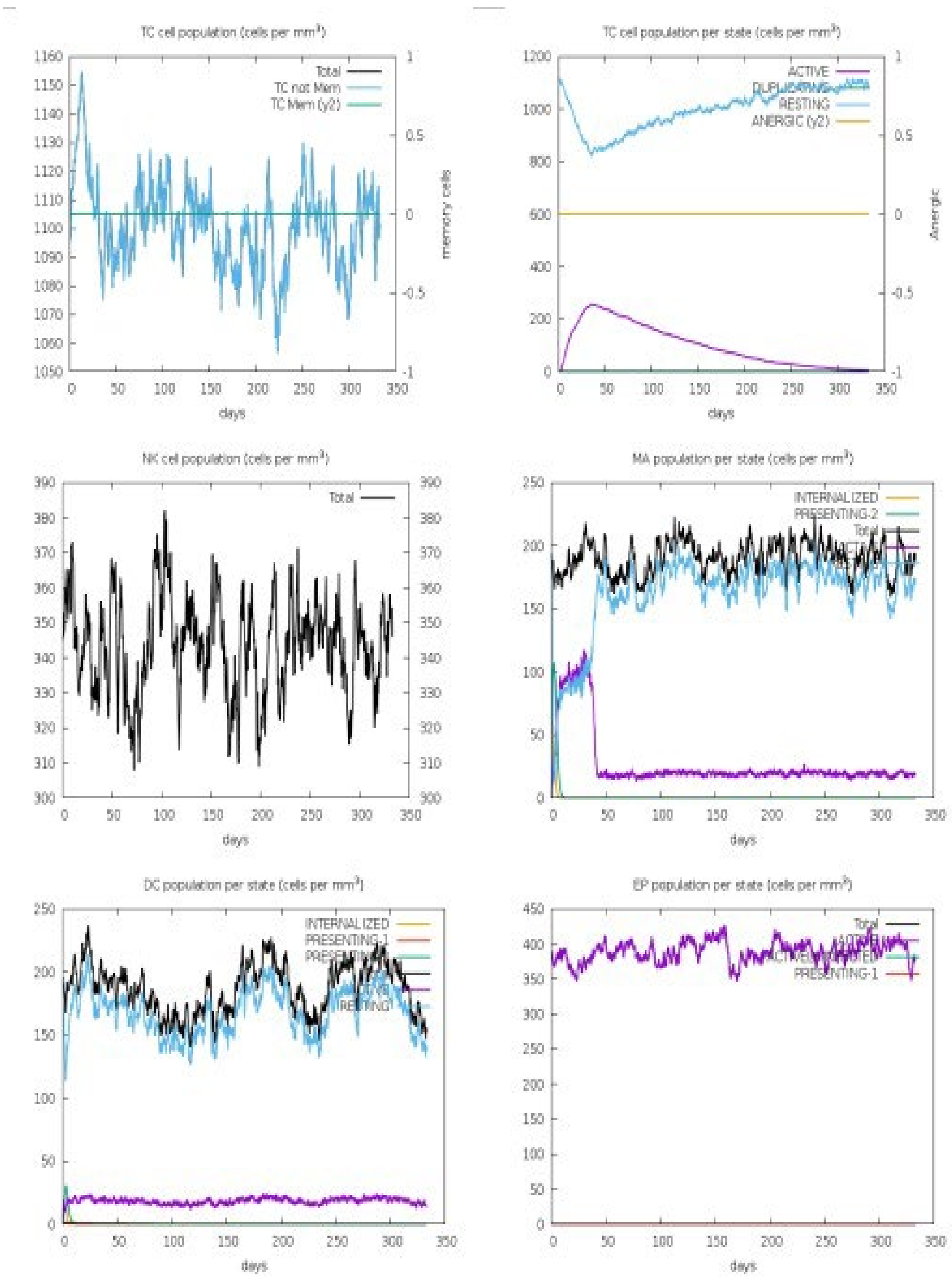

Figure S2 T-cell responses to the vaccine construct. T_c (cytotoxic killer T-cell), macrophages (Mφ), natural killer cell, dendritic and epithelial cell.
